# Characterization of the oxidative stress stimulon and PerR regulon of *Campylobacter jejuni*

**DOI:** 10.1186/1471-2164-10-481

**Published:** 2009-10-18

**Authors:** Kiran Palyada, Yi-Qian Sun, Annika Flint, James Butcher, Hemant Naikare, Alain Stintzi

**Affiliations:** 1 Department of Veterinary Pathobiology, College of Veterinary Medicine, Oklahoma State University, Stillwater, OK 74078, USA; 2 Ottawa Institute of Systems Biology, Department of Biochemistry, Microbiology and Immunology, Faculty of Medicine, University of Ottawa, 451 Smyth road, Ottawa, ON, K1H 8M5, Canada

## Abstract

**Background:**

During gut colonization, the enteric pathogen *Campylobacter jejuni *must surmount the toxic effects of reactive oxygen species produced by its own metabolism, the host immune system, and intestinal microflora. Elucidation of *C. jejuni *oxidative stress defense mechanisms is critical for understanding *Campylobacter *pathophysiology.

**Results:**

The mechanisms of oxidative stress defense in *C. jejuni *were characterized by transcriptional profiling and phenotypic analysis of wild-type and mutant strains. To define the regulon of the peroxide-sensing regulator, PerR, we constructed an isogenic Δ*perR *mutant and compared its transcriptome profile with that of the wild-type strain. Transcriptome profiling identified 104 genes that belonged to the PerR regulon. PerR appears to regulate gene expression in a manner that both depends on and is independent of the presence of iron and/or H_2_O_2_. Mutation of *perR *significantly reduced motility. A phenotypic analysis using the chick colonization model showed that the Δ*perR *mutant exhibited attenuated colonization behavior. An analysis of changes in the transcriptome induced by exposure to H_2_O_2_, cumene hydroperoxide, or menadione revealed differential expression of genes belonging to a variety of biological pathways, including classical oxidative stress defense systems, heat shock response, DNA repair and metabolism, fatty acid biosynthesis, and multidrug efflux pumps. Mutagenic and phenotypic studies of the superoxide dismutase SodB, the alkyl-hydroxyperoxidase AhpC, and the catalase KatA, revealed a role for these proteins in oxidative stress defense and chick gut colonization.

**Conclusion:**

This study reveals an interplay between PerR, Fur, iron metabolism and oxidative stress defense, and highlights the role of these elements in *C. jejuni *colonization of the chick cecum and/or subsequent survival.

## Background

*Campylobacter *infection is an acute diarrheal disease with symptoms that range from a day of mild watery or bloody diarrhea to severe abdominal pain that can last for several weeks [1]. This pathogen has been implicated in the development of Guillain-Barré syndrome and is a suspected etiological factor in Crohn's disease and ulcerous colitis [2-4].

*Campylobacter jejuni *is a microaerophilic bacterium and therefore requires reduced oxygen for growth [5]. Thus, during the course of normal metabolism, *C. jejuni *will unavoidably be exposed to reactive oxygen species (ROS) [6]. These ROS, which include superoxide radicals (O_2_^·-^), hydrogen peroxide (H_2_O_2_) and hydroxyl radicals (·OH), are formed by the stepwise one-electron reduction of molecular oxygen [6]. During aerobic metabolism, the auto-oxidation of respiratory dehydrogenases generates superoxide radicals and H_2_O_2 _[7]. When cellular iron is present, ferrous iron catalyzes the transfer of an electron to H_2_O_2_, generating a hydroxyl radical (·OH), the strongest oxidant that exists in aqueous environments [6]. In addition, *C. jejuni *are exposed to ROS produced by the host immune system and by the host intestinal microflora [8-12]. Irrespective of their source, ROS damage DNA and proteins, and cause peroxidation of lipids [6,7]. In order to survive, microorganisms have developed specialized and inducible defense mechanisms to protect against oxidative stress [13].

Analyses of the sequenced *C. jejuni *genomes reveal the presence of a number of enzymes and proteins that are thought or known to protect *Campylobacter *against the damaging effects of oxidative stress. The roles of SodB (superoxide dismutase), KatA (catalase), AhpC (alkyl-hydroxyperoxidase), Cft (ferritin), FdxA (ferredoxin), and Dps (DNA-binding protein from starved cells) in *C. jejuni *oxidative stress defense mechanisms have been partially characterized [14]. Previous reports have shown that AhpC and SodB are required for *C. jejuni *survival in aerobic conditions [15,16], and that *C. jejuni katA *and *sodB *mutants have a reduced ability to survive within macrophages and INT407 cells, respectively [16,17]. In addition, a *sodB *mutant was recently shown to exhibit increased sensitivity to paraquat exposure compared to the wild-type strain [18]. These observations suggest that KatA and SodB directly contribute to *Campylobacter *pathogenicity and oxidative stress defense. Both *cft*- and *dps*-deficient mutants of *C. jejuni *were found to be more sensitive to H_2_O_2 _than were their parent strains [19,20]. An *fdxA*-deficient mutant exhibited significantly reduced aerotolerance, but its resistance to H_2_O_2 _and cumene hydroperoxide were unaffected [21]. The specific function of FdxA in oxidative stress defense remains to be elucidated. In addition, the roles of *C. jejuni *SodB, KatA, AhpC, Cft, Dps, and FdxA in vivo during gut colonization and infection have not been reported.

In many enteric Gram-negative bacteria, the oxidative stress defense mechanisms are regulated by the superoxide- and peroxide-sensing regulators, SoxRS and OxyR, respectively [7]. These regulators are absent from the *C. jejuni *genome, which instead possesses the peroxide-sensing regulator, PerR [22]. Inactivation of PerR results in constitutive expression of KatA and AhpC, and enhanced resistance to peroxide stress [22]. By analogy with *B. subtilis *PerR [23], the *C. jejuni *regulator is presumed to be a zinc-containing metalloprotein that requires either iron or manganese as a regulatory metal ion. Biochemical studies have recently demonstrated that *Bacillus subtilis *PerR senses H_2_O_2 _by Fe^2+^-catalyzed oxidation of two histidines in its regulatory binding site, which leads to Fe^2+ ^release and subsequent de-repression of PerR target genes [23]. If, instead of Fe^2+^, Mn^2+ ^is bound to the regulatory site, the resulting Mn^2+^-PerR complex is insensitive to oxidative inactivation [23]. In addition to PerR, the *C. jejuni *ferric uptake regulator, Fur, is also involved in the direct regulation of *katA*, as demonstrated by the observation that the *fur *gene must also be mutated to fully abolish iron-dependent *katA *regulation in a *perR *mutant [22]. This observation indicates that, although the Fur regulon is primarily composed of genes encoding proteins involved in iron metabolism [24], there is at least some overlap between the PerR and Fur regulons. Notably, whereas Fur is indirectly involved in the regulation of *sodB *in *E. coli *via the small-RNA, *ryhB *[25], there is currently no evidence for *ryhB*-like regulation in *C. jejuni*. Genome-wide transcriptional analyses have revealed that *C. jejuni *Fur regulates several proteins with potential functions in oxidative stress defenses, including KatA, FdxA, and TrxB [24,26]. The Fur protein is a homodimer that, upon binding its co-repressor Fe^2+^, binds to a consensus sequence in the promoter of Fur-regulated genes, repressing their transcription [27,28]. Clearly, available evidence suggests an interconnection between *C. jejuni *iron metabolism and oxidative stress defense. As a first step toward understanding the interplay between Fur, PerR, oxidative stress and iron homeostasis, we used transcriptional profiling and mutagenesis studies to define the role of PerR in regulating the oxidative stress stimulons of *C. jejuni*.

## Results and Discussion

### Construction and phenotypic characterization of a Δ*perR C. jejuni *mutant

To assess the physiological function and regulatory role of PerR, we disrupted the *perR *gene in *C. jejuni *NCTC 11168 by deletion of 69% of the coding sequence and insertion of a chloramphenicol resistance cassette, as described in Materials and Methods. In *C. jejuni *NCTC 11168, the *perR *gene is located upstream of a gene encoding the hypothetical protein, Cj0323. To rule out the possibility of a polar mutation, we complemented the Δ*perR *mutant with a wild-type copy of the *perR *gene, inserted at the rRNA locus, as described by others [29]. The Δ*perR *mutant and the wild-type strain exhibited similar growth properties (data not shown). To determine the role played by PerR in modulating oxidative stress resistance, we assessed the phenotype of the Δ*perR *mutant and *perR*-complemented strain by disk inhibition assay [15,30], using three oxidants: H_2_O_2_, cumene hydroperoxide, and menadione. As expected, the Δ*perR *mutant was more resistant toH_2_O_2 _and cumene hydroperoxide than was the wild-type strain (Table 1). This observation is in agreement with the role of PerR as a repressor of *katA *and *ahpC *expression, and is in accordance with previously published results [22]. In addition, the Δ*perR *mutant was more sensitive to menadione than was the wild-type strain. This observation, which has not been reported previously, suggests that PerR might activate genes involved in menadione resistance. The oxidative stress sensitivity of the wild type was restored in the *perR*-complemented strain, confirming that the phenotype of the Δ*perR *mutant results solely from the disruption of the *perR *gene.

**Table 1:** Oxidative stress sensitivity of *C. jejuni* NCTC 11168 mutants containing mutations of *perR*, *fur*, *ahpC*, *katA*, or *sodB* genes, and the corresponding complemented strains determined by disk diffusion assays

**Strain**	**H_2_O_2_**	**Cumene hydroperoxide**	**Menadione**
*C. jejuni *NCTC 11168	19.08 ± 0.19	24.50 ± 0.22	29.39 ± 0.53
Δ*perR*	6.00 ± 0.00	20.93 ± 0.52	33.90 ± 1.98
Δ*perR *+ *perR*	17.61 ± 0.15	21.89 ± 0.40	30.28 ± 0.24
Δ*fur*	16.73 ± 0.32	27.53 ± 0.64	31.60 ± 1.55
Δ*fur *+ *fur*	20.00 ± 0.25	25.23 ± 0.99	31.08 ± 1.37
Δ*perR*Δ*fur*	7.89 ± 0.11	22.28 ± 0.36	35.56 ± 0.20
Δ*ahpC*	17.23 ± 0.50	34.87 ± 0.79	29.05 ± 1.81
Δ*ahpC *+ *ahpC*	18.45 ± 0.10	25.40 ± 0.34	28.38 ± 0.39
Δ*katA*	28.85 ± 0.87	26.43 ± 0.47	29.85 ± 1.39
Δ*katA *+ *katA*	10.83 ± 1.24	23.83 ± 0.17	31.00 ± 1.20
Δ*sodB*	22.10 ± 0.35	30.75 ± 3.53	38.28 ± 1.96
Δ*sodB *+ *sodB*	21.78 ± 0.33	25.40 ± 0.17	37.50 ± 2.62

The data represent the diameters (in millimeters) of the clear zones. Data shown are the means ± standard errors of at least triplicate experiments. The diameter of the disk is 6 mm, and each disk contained 10 μL of H_2_O_2 _(3%), cumene hydroperoxide (3% in DMSO), or menadione sodium bisulfite (90 mM in H_2_O).

### Microarray experimental design

Genes encoding proteins involved in oxidative stress defense are known to be induced in response to reactive oxygen species [7]. PerR has been previously shown to repress the transcription of two genes, *katA *and *ahpC*, and is thought to be the major peroxide stress regulator in *C. jejuni *[22]. To characterize the genes regulated by oxidants and/or PerR in *C. jejuni *NCTC 11168, we performed three sets of microarray experiments.

**Figure 1:**
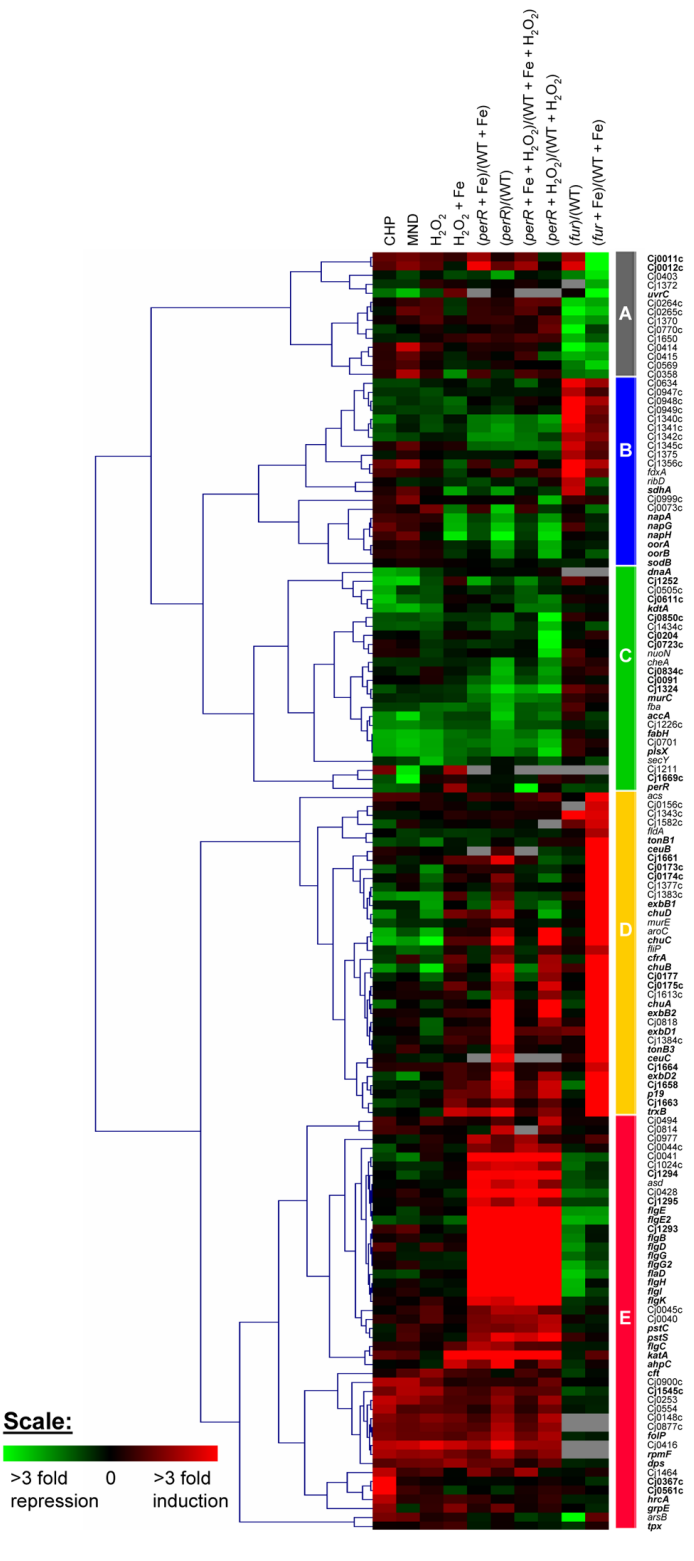
**Hierarchical clustering analysis of genes affected by oxidant or found to be regulated by PerR or Fur**. *From left to right*: the first four columns represent changes in the transcriptome of the wild-type *C. jejuni *strain grown in MEMα medium in response to a 10-minute exposure to cumene hydroperoxide (labeled CHP), menadione (labeled MND), hydrogen peroxide (labeled H_2_O_2_), and H_2_O_2 _in the presence of iron (labeled H_2_O_2 _+ Fe). The next four columns represent the changes in transcript level in the wild-type strain compared to the Δ*perR *mutant grown in MEMα medium determined 15 minutes after the addition of ferrous sulfate (labeled [*perR *+ WT]/[WT + Fe]), in MEMα medium (labeled *perR*/WT), 15 minutes after the addition of ferrous sulfate followed by a 10-minute exposure to H_2_O_2 _(labeled [*perR *+ Fe + H_2_O_2_]/[WT + Fe + H_2_O_2_]), and 10 minutes after exposure to H_2_O_2 _(labeled [*perR *+ H_2_O_2_]/[WT + H_2_O_2_]). The last two columns represent the changes in transcript levels in the wild-type *C. jejuni *strain compared to the Δ*fur *mutant grown to mid-log phase in MEMα medium (labeled *fur*/WT) and 15 minutes after addition of ferrous sulfate (labeled [*fur *+ Fe]/[WT +Fe]). The Fur regulon was previously reported in Palyada *et al. *[24]. The intensity of red and green indicates the degree of change, as represented by the scale at the bottom. Genes in boldface are further discussed in the text.

The first set of experiments sought to define the PerR regulon. To identify the genes regulated by PerR, we compared transcript levels in the Δ*perR *mutant with those in the parental wild-type strain using microarray analyses. Four different experimental conditions were examined. First, we grew the Δ*perR *mutant and wild-type strain to mid-log phase in iron-restricted minimal essential medium alpha (MEMα) and, after adding 40 μM ferrous sulfate and incubating for 15 minutes, compared Δ*perR *mutant and wild-type transcriptional profiles (Figure 1, column labeled [*perR *+ Fe]/[WT + Fe]). Because PerR is a repressor in the presence of iron, genes with higher transcript levels in the Δ*perR *mutant than in the wild-type strain are possible members of the PerR regulon. Second, we compared the transcriptomes of the Δ*perR *mutant and wild-type strain grown to mid-log phase in iron-restricted MEMα (Figure 1, column labeled *perR*/WT). This experimental condition should reveal genes regulated by PerR in the absence of iron. Third, we grew the Δ*perR *mutant and wild-type strain to mid-log phase in iron-restricted MEMα and compared their transcriptomes after adding 1 mM H_2_O_2 _and incubating for 10 minutes (Figure 1, column labeled [*perR *+ H_2_O_2_]/[WT + H_2_O_2_]). This comparison should reveal genes regulated by PerR in response to H_2_O_2 _and/or in the absence of added iron. Fourth, we compared the transcriptomes of the Δ*perR *mutant and wild-type strain grown to mid-log phase in iron-restricted MEMα after supplementing with 40 μM ferrous sulfate (15-minute incubation) and then adding H_2_O_2 _(10-minute incubation) (Figure 1, column labeled [*perR *+ Fe + H_2_O_2_]/[WT + Fe + H_2_O_2_]). Because PerR-mediated repression in the presence of iron is relieved by H_2_O_2_, this comparison should reveal PerR-regulated genes that are unresponsive to H_2_O_2 _exposure in the presence of iron.

In the second set of experiments, designed to define the hydrogen peroxide stimulon, we monitored the transcriptional profiles of *C. jejuni *in response to a 15-minute exposure to 1 mM H_2_O_2 _in the presence of iron, as described in Materials and Methods. Because PerR represses genes using iron as a co-repressor, genes that are de-repressed by the addition of H_2_O_2 _are possible members of the PerR regulon.

The objective of the third set of experiments was to identify genes that are responsive to oxidant exposure in the absence of iron. Here, we studied *Campylobacter *gene expression in iron-restricted cells in response to a 10-minute exposure to three classes of reactive oxygen species (each at a final concentration of 1 mM): H_2_O_2_, cumene hydroperoxide (an organic hydroperoxide), and menadione (a superoxide generator). Preliminary experiments showed that *C. jejuni *remained viable during this 10-minute exposure to reactive oxygen species (data not shown), indicating that the observed transcriptional changes correspond to *C. jejuni *responses to a sub-lethal concentration of oxidants. Under these iron-limited conditions, PerR-repressed genes should be highly expressed and unresponsive to oxidant exposure. Therefore, any genes that respond to oxidant(s) in an Fe^2+^/PerR-independent fashion should be revealed by these transcriptome analyses.

### Global transcriptome analyses

To characterize the PerR regulon and oxidant stimulons, we merged the microarray data for the three sets of experiments and performed a hierarchical clustering analysis (Figure 1 and Additional file 1). Because Fur is known to co-regulate several oxidative stress defense genes (including *katA*, *fdxA *and *trxB*) and control the acquisition of iron (the co-repressor of PerR), we also included the expression profiles of previously described members of the Fur regulon in the analysis [24]. Genes were included in the cluster analysis if their change in transcript abundance was ≥ 2 fold with a *P *value ≤ 10^-3 ^in at least one experimental condition. In addition, all known oxidative stress defense genes were also included, even if their expression profiles did not meet the fold-change and *P*-value thresholds. The validity of the microarray data was confirmed by quantitative reverse-transcriptase PCR (qRT-PCR) for five genes: *cft*, *perR*, *ahpC*, *katA*, and *sodB*. The *cft *gene was found to be 6-fold up-regulated in response to H_2_O_2 _exposure under iron-restricted conditions, whereas the transcript level of *perR *was unchanged. These fold changes correlated with the microarray data. The fold changes in *ahpC*, *katA *and *sodB *expression were evaluated in response to exposure to cumene hydroperoxide, menadione and H_2_O_2_, with and without iron (Table 2). qRT-PCR revealed that KatA, AhpC, and SodB mRNA levels were significantly up-regulated in response to H_2_O_2 _in the presence of iron, and the qRT-PCR-determined fold changes in these genes were well correlated with the microarray data. Notably, although qRT-PCR analyses found that *ahpC, katA*, and *sodB *were also significantly up-regulated in response to oxidant exposure under iron-restricted conditions, microarray techniques did not identify these genes as being differentially expressed under these conditions (Table 2). The failure of the microarray experiments to identify *ahpC, katA*, and *sodB *up-regulation can be explained by the fact that these three genes were highly expressed under iron-restricted conditions, resulting in saturation of the microarray hybridization signals. Thus, because the level of transcripts that hybridize to the DNA on the microarray spot reached the maximum binding capacity of the printed DNA, the expected increase in the expression of these genes upon oxidant exposure could not be accurately determined.

**Table 2:** qRT-PCR analysis of *katA*, *ahpC*, and *sodB *gene expression.

	**Fold change in response to**			
**Gene**	**Cumene hydroperoxide**	**Menadione**	**H_2_O_2_**	**H_2_O_2_+ Fe**
*sodB*	30.8 ± 1.5	12.2 ± 1.6	2.3 ± 1.7	4.2 ± 0.1
*ahpC*	4.3 ± 1.3	2.3 ± 0.1	2.2 ± 0.1	9.2 ± 0.4
*katA*	6.7 ± 1.3	5.4 ± 0.2	5.8 ± 0.2	132 ± 7.2
*perR*	nd^a^	nd^a^	1.3 ± 0.1	2.0 ± 0.1

^a^nd, not determined

Data are presented as the mean change in expression ± standard deviation for each gene.

A total of 143 genes were differentially regulated by a minimum of 2-fold under at least one of the ten experimental conditions tested (corresponding to the ten columns in Figure 1). The cluster analysis identified five major clusters: A, B, C, D, and E. This analysis showed that the majority of the oxidant responsive genes belong to the PerR and/or Fur regulons, suggesting that PerR and Fur are the main regulators of the oxidative stress response in *C. jejuni*. Importantly, this clustering also revealed a subset of genes from the PerR regulon that were not responsive to oxidant exposure, indicating a regulatory role for PerR beyond its classical regulation of oxidative stress genes.

**Figure 2:**
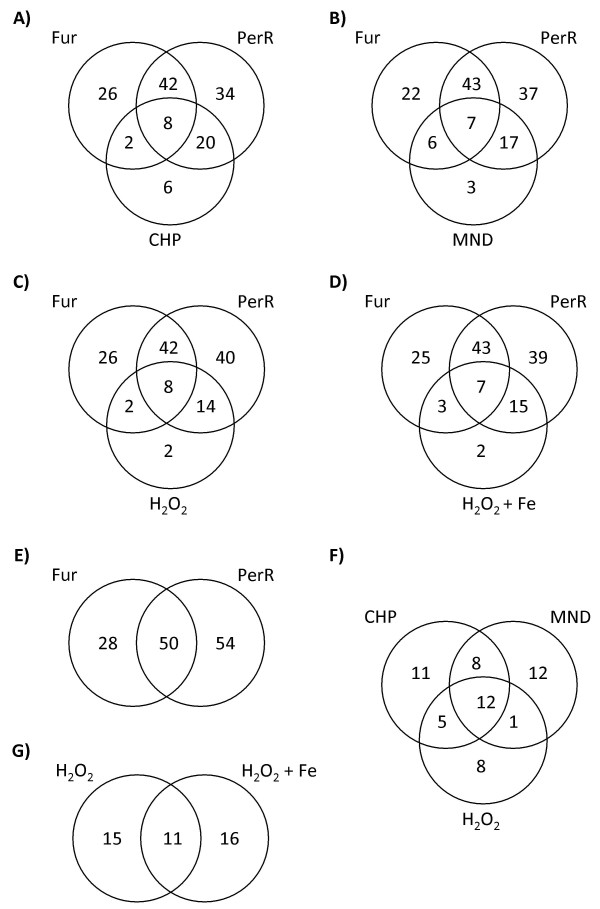
**Venn diagrams showing overlap among *C. jejuni *genes from the menadione (MND), cumene hydroperoxide (CHP), hydrogen peroxide (H_2_O_2_), and hydrogen peroxide-plus-iron stimulons, and the PerR and Fur regulons**. The numbers in the Venn diagram indicate the number of genes. (A) Venn diagram showing overlap among genes from the Fur and PerR regulons, and the cumene hydroperoxide stimulon. (B) Venn diagram showing overlap among genes from the Fur and PerR regulons, and the menadione stimulon. (C) Venn diagram showing overlap among genes from the Fur and PerR regulons, and the hydrogen peroxide stimulon. (D) Venn diagram showing overlap among genes from the Fur and PerR regulons, and the hydrogen peroxide-plus-iron stimulon. (E) Venn diagram showing overlap among genes from the Fur and PerR regulons. (F) Venn diagram showing overlap among genes from the menadione, cumene hydroperoxide, and hydrogen peroxide stimulons. (G) Venn diagram showing overlap between the hydrogen peroxide and hydrogen peroxide-plus-iron stimulons.

To further analyze logical relationships (unions and intersections) of genes under each of the different conditions, we performed a series of Venn diagram analyses (Figure 2). Genes were assigned to a particular stimulon (cumene hydroperoxide, menadione, or H_2_O_2_) or regulon (Fur or PerR) if their expression ratio changed > 1.5 fold under the corresponding conditions. These diagrams provide an important overview, showing the overlaps between the Fur and PerR regulons, and the hydrogen peroxide, cumene hydroperoxide and menadione stimulons (Figure 2). The Fur and PerR regulons are composed of at least 78 and 104 genes, respectively; 50 of these genes are co-regulated (Figure 2E and Additional files 2 and 3). The cumene hydroperoxide, menadione, and hydrogen peroxide stimulons are composed of at least 36, 33, and 26 genes, respectively (Figure 2F and Additional files 4, 5, 6). Of these genes, 11, 12, and 8 are unique to the cumene hydroperoxide, menadione, and hydrogen peroxide stimulons, respectively, and 12 belong to all three stimulons.

These data indicate that some of the transcriptome changes are oxidant specific. Moreover, the class of genes responsive to H_2_O_2 _exposure is dependent on the level of iron. Indeed, the differential expression of 16 H_2_O_2_-responsive genes was found to strictly depend on the presence of iron, whereas the expression of 15 genes was only affected under iron-restricted conditions (Figure 2G and Additional file 7). Additionally, the expression of 11 genes was found to be responsive to H_2_O_2 _exposure independent of the presence of iron. PerR appears to be the major regulator of oxidative stress responses, regulating 74% of genes belonging to the cumene hydroperoxide stimulon, 73% of the genes in the menadione stimulon, and 85% of the genes in the hydrogen peroxide stimulon (with or without iron). Although Fur also appears to regulate genes belonging to the oxidant stimulons, most of the Fur-regulated genes were co-regulated by PerR. Nevertheless, Fur regulated 28% of the genes from the cumene hydroperoxide stimulon, 30% of the genes from the menadione stimulon, and 38% of the genes from the hydrogen peroxide stimulon (with or without iron).

### The PerR regulon in *C. jejuni*

PerR has previously been shown to repress the transcription of two genes, *katA *and *ahpC *[22]. Here, we show by genome-wide transcriptional profiling that the PerR regulon contains at least 104 genes (Figures 1 and 2). The hierarchical clustering analysis clearly grouped the PerR-repressed genes into clusters E and D, and the PerR-activated genes into clusters B and C (Figure 1). Genes belonging to these clusters will be discussed in more detail below. It is important to note, however, that these microarray experiments cannot distinguish between direct and indirect effects of PerR on gene expression.

Cluster E is composed of 46 transcripts that were repressed by PerR. Genes from cluster E could be divided in 2 groups: (1) genes whose transcript levels increased in response to exposure to at least one of the oxidants (Figure 1; columns labeled CHP, MND, H_2_O_2_, and H_2_O_2 _+ Fe), and (2) genes whose transcript levels were unaffected or were slightly decreased by the addition of an oxidant. As expected, genes that have been previously shown to play a role in oxidative stress defense (or have been annotated as such) belong to the first group. These genes encode catalase (KatA), alkyl hydroxyl-peroxidase (AhpC), ferritin (Cft), and a DNA-binding protein from starved cells (Dps). Interestingly, these genes were repressed in the wild-type strain, even under iron-restricted conditions, despite the requirement of iron for PerR repression. This result is in agreement with the up-regulation of these genes in response to oxidant exposure under iron-restricted conditions (Figure 1; columns labeled CHP, MND and H_2_O_2_, and Table 2). The responsiveness of these genes to oxidant exposure under iron-restricted conditions might be explained by trace amounts of iron in the growth medium or other divalent cations that can replace iron to facilitate a slight PerR repression[28].

One gene of particular interest in cluster E is *Cj1545c*, an *mdaB *homolog. Recent studies have shown that a *Helicobacter pylori mdaB *mutant is sensitive to H_2_O_2_, organic hydroperoxides, and the superoxide-generating agent, paraquat [31]. MdaB is an NADPH quinone reductase capable of reducing quinone to quinol using NADPH as the preferred electron acceptor [31]. MdaB is thought to protect *H. pylori *cells from oxidative stress by competing with the quinone one-electron reduction pathway, thus preventing the production of semiquinone radicals, which form superoxide radicals by reacting with molecular oxygen [31,32]. Because MdaB is up-regulated in *C. jejuni *upon exposure to each of the three oxidants and is regulated by PerR, it is tempting to speculate that MdaB might be similarly important in oxidative stress management in *C. jejuni*.

Other genes encoding proteins with known or potential functions that are PerR-repressed and oxidant-induced include *folP *(encoding a probable dihydropteroate synthase), *pstC *and *pstS *(encoding components of a probable phosphate transporter), and *rpmF *(encoding a ribosomal protein). The up-regulation of the ribosomal protein, RpmF, is intriguing. In *Mycobacterium smegmatis*, overproduction of the ribosomal protein, S13, has been shown to enhance catalase and peroxidase activities [33]. A similar role for RpmF in *C. jejuni *has not yet been demonstrated. Similarly, the functions of these other differentially expressed genes in oxidative stress defense remain to be investigated.

**Figure 3:**
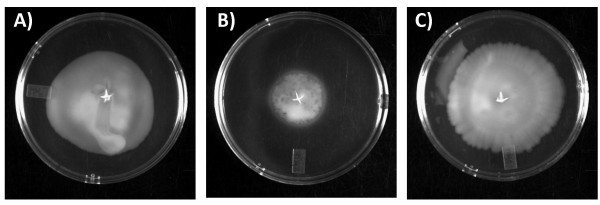
**Motility assay of the wild-type *C. jejuni *NCTC 11168 strain (A), the Δ*perR *mutant (B), and the complemented Δ*perR+perR *strain (C) on MH plates containing 0.4% agar**.

Another prominent group of genes in cluster E--*flgD, flaD, flgE, flgG, flgE2, flgG2, flgH, flgI, flgB, flgK, flgC, Cj1293 (pseB), Cj1294 (pseC) *and *Cj1295*--encode proteins involved in flagellum biogenesis. The direct or indirect regulation of this set of genes by PerR suggests that oxidative stress might impair *Campylobacter *motility. While these genes were not differentially expressed in response to exposure to sub-lethal concentrations of the three oxidants tested, they might be affected under more severe stress conditions. Interestingly, the expression of these genes was previously shown to be repressed by iron and activated by apo-Fur (Figure 1 and [24]), suggesting that the expression of these genes is directly or indirectly regulated by both Fur and PerR. To assess the role of PerR in motility behavior, we determined the motility of the wild-type strain, the Δ*perR *mutant, and the complemented Δ*perR+perR *strain in semi-solid motility plates. Mutation of *perR *significantly reduced motility, whereas complementation of Δ*perR+perR *in trans fully restored wild-type motility (Figure 3). The phenotype of the Δ*perR *mutant suggests that disregulation of flagellum genes results in impaired motility and provides additional evidence that PerR regulates genes beyond the classical oxidative stress regulon.

Cluster D is composed of 36 genes whose transcript levels were increased in the Δ*perR *mutant compared to the wild-type strain under conditions of iron restriction. The direct or indirect PerR repression of several of these genes could be partially relieved by the addition of H_2_O_2_. It is important to note that the genes in cluster D are also Fe^2+^/Fur-repressed, consistent with the fact that a majority of these genes encode proteins involved in iron-acquisition systems. These include the heme transporter system (ChuABCD), the ferric-enterobactin transporter (CfrA and CeuC), components of the energy-transducing TonB-ExbB-ExbD complexes (TonB3, ExbB2, ExbD2, ExbB1, and ExbD1), proteins potentially involved in iron acquisition from transferrin (Cj0177 [ChaN] and Cj0173c-175c [CfpbABC]), and an additional potential iron-acquisition system (p19, Cj1661, Cj1663, Cj1664 and Cj1658). The up-regulation of this set of genes in the Δ*perR *mutant (Figure 1, columns labeled *perR*/WT and [*perR *+ H_2_O_2_]/[WT + H_2_O_2_]) might be an indirect consequence of the Δ*perR *mutation.

Indeed, over-expression of iron and heme-containing proteins, including KatA and Dps, in the Δ*perR *mutant might further deplete the cells of iron, relieving Fur repression and leading to the observed gene expression profile. Taken together, the regulatory profile of the iron-acquisition genes highlights the close interlink between oxidative stress and iron metabolism. In fact, these iron-acquisition systems were found to be down-regulated in response to oxidant exposure under iron-restricted conditions (Figure 1; columns labeled CHP, MND, and H_2_O_2_). This down-regulation might indirectly contribute to cell survival by preventing further iron-dependent ROS production catalyzed by the uptake of additional iron. Moreover, the repression of these genes by an oxidant might be an indirect consequence of iron released from protein iron-sulfur clusters, such as aconitase and fumarase, which are known to be highly susceptible to oxidative degradation [7]. Undoubtedly, an increase in free iron in the cytoplasm would result in down-regulation of the genes belonging to the Fur regulon. Interestingly, H_2_O_2 _exposure in the presence of excess iron slightly induced the expression of several of these genes, including the heme transporter operon (Figure 1, column labeled H_2_O_2 _+ Fe). The differential expression of the heme transporter is surprising as it is difficult to envision increased iron uptake under iron-replete, H_2_O_2_-stress conditions. It is more likely that up-regulation of the heme transporter might result in the accumulation of intracellular heme. Heme is known to exhibit antioxidant properties by scavenging reactive oxygen species, and can also replace damaged heme centers degraded by H_2_O_2 _[34]. In addition, given that catalase requires heme as a cofactor for its activity [35], it is tempting to speculate that the increased uptake of heme is required to generate or maintain an active pool of catalase. Moreover, because the addition of iron has been previously shown to drastically repress *chuABCD *genes [24], the up-regulation of these genes by H_2_O_2 _suggests that H_2_O_2 _negates iron repression. In *E. coli*, submicromolar concentrations of H_2_O_2 _have been shown to de-repress the Fur regulon, likely by oxidizing the Fe^2+^-Fur complex [36]. However, a similar deactivation of the Fe^2+^-Fur complex in *C. jejuni *in response to H_2_O_2 _remains to be demonstrated.

As shown in Figure 1, PerR-activated genes were grouped into clusters B and C. Cluster C contains 25 genes; the transcript levels of 22 of these genes were decreased in the Δ*perR *mutant compared to the wild-type strain. The expression of these genes was also repressed by the addition of oxidant. Taken together, the expression profiles of these genes suggest that they might be PerR-activated. It should also be noted that genes in cluster C were not Fur-regulated.

Cluster C contains a number of genes encoding proteins involved in fatty acid biosynthesis that were down-regulated by oxidant exposure and activated by PerR. These include the *accA *gene, encoding a subunit of the acetyl-CoA carboxylase; *fabH*, encoding a probable 3-oxoacyl-(acyl-carrier-protein) synthase; and *plsX*, encoding a putative fatty acid/phospholipid synthesis protein. The down-regulation of these genes might indicate growth arrest. Alternatively, free radicals are known to initiate lipid peroxidation chain reactions yielding highly genotoxic radicals, such as lipid hydroperoxides, lipid radicals, peroxyl radicals, and peroxyl radicals [37]. Thus, the down-regulation of these genes, taken together with the differential expression of numerous genes encoding membrane proteins and proteins involved in the biogenesis of capsule and other membrane structures (Cj1252, Cj0611c, Cj0850c, Cj0204, Cj0723c, Cj834c, Cj0091, Cj1324, *murC *and *kdtA*), could also indicate a remodeling of the outer-membrane and cell wall structures in response to oxidative stress. The extent and significance of this membrane-structure remodeling remains to be investigated. Nevertheless, a similar response was also observed in *Rhodobacter sphaeroides *cells exposed to H_2_O_2 _[38]. Notably, PerR belongs to cluster C and was found to directly or indirectly activate its own transcription in the presence of iron and H_2_O_2 _(Figure 1, column labeled [*perR *+ Fe + H_2_O_2_]/[WT + Fe + H_2_O_2_]). Although the Δ*perR *mutant was constructed by deleting 69% of the *perR *coding sequence, the 60 base pairs remaining in the 5' end allow determination of the *perR *expression profile by our microarray. This autoregulation of *perR *transcription correlates with the up-regulation of PerR mRNA in the wild-type strain in the presence of H_2_O_2 _and iron (Figure 1, column labeled H_2_O_2 _+ Fe). This up-regulation of *perR *expression was confirmed by qRT-PCR, which demonstrated approximately a 2-fold increase in PerR mRNA (Table 2). The significance of this regulation is unclear but might be a consequence of the overlap between the PerR and Fur regulons. The function of the other PerR-activated genes in oxidative stress defenses and/or *Campylobacter *physiology requires further investigation.

Cluster B contains 21 genes, most of which are positively transcriptionally regulated by PerR and/or repressed by Fur. Genes encoding proteins involved in energy metabolism (*napAGH *and *oorAB*) were activated by PerR, slightly induced in response to cumene hydroperoxide and menadione exposure, and repressed in response to exposure to H_2_O_2 _in the presence of iron. Transcripts of genes that encode proteins containing iron-sulfur clusters or iron, including NapA (nitrite reductase), NapG and NapH (ferredoxins), and OorAB (2-oxoglutarate oxidoreductase), were decreased in the Δ*perR *mutant compared to the wild-type strain under iron-restricted conditions. As previously noted, this regulation could be an indirect consequence of a more severe iron scarcity caused by the *perR *mutation. Indeed, these genes belong to the iron stimulon and have been shown to be preferentially expressed under iron-replete conditions [24]. Nevertheless, direct regulation of these genes by PerR could not be precluded; in fact, their down-regulation in response to H_2_O_2 _in the presence of iron might prevent the formation of endogenous hydroxyl radicals. Indeed, aerobic respiratory chains are known to be a significant source of endogenous reactive oxygen species. Another cluster B gene of interest is *sodB*, which codes for superoxide dismutase. The level of SodB transcript appeared to be increased in the wild-type strain compared to the Δ*perR *mutant under conditions of iron restriction (with or without H_2_O_2_). The high levels of *sodB *expression under iron-restricted conditions resulted in the saturation of the hybridization signals, preventing microarray experiments from accurately measuring *sodB *expression. Therefore, we monitored *sodB *expression by qRT-PCR. As shown in Table 2, *sodB *expression was significantly increased in response to oxidant exposure, particularly in the presence of cumene hydroperoxide and the superoxide generator, menadione which is consistent with a role involving the detoxification of superoxide anions. Other genes from cluster B encode proteins of uncharacterized function.

Finally, cluster A is composed of 14 genes that are mainly Fur-activated. Only two genes from this cluster, *Cj0011c *and *Cj0012c *(*rrc*), appear to be PerR repressed. *Cj0011c *encodes a DNA-binding protein involved in natural transformation [39]. *Cj0012c *(*rrc*) encodes a protein homologous to rubredoxin oxidoreductase/rubrerythrin proteins, which have been shown to protect anaerobic microorganisms against oxidative stress by scavenging ROS through an oxidation-reduction reaction involving bound iron [40,41]. Recently, the amount of Cj0012c protein in *C. jejuni *has been reported to decrease in response to H_2_O_2 _exposure due to degradation of the protein [41]. Consequently, it is tempting to propose that the up-regulation of the Cj0012c transcript might compensate for the enhanced sensitivity of the protein to oxidative stress.

### Effects of iron on the hydrogen peroxide stimulon

To evaluate the effect of iron on the *C. jejuni *oxidative stress response, we studied transcriptional changes in *C. jejuni *after a 10-minute exposure to H_2_O_2 _in the presence and absence of iron (Figure 1, columns labeled H_2_O_2 _+ Fe and H_2_O_2_). A Venn diagram analysis identified 11 differentially expressed genes that were common to both treatments, and 15 and 16 genes whose transcriptional profiles were uniquely altered by H_2_O_2 _in the absence and presence of iron, respectively (Figure 2). The effect of iron on gene expression is likely an indirect consequence of its role as a co-repressor of PerR and a catalytic activator of the Fenton reaction. Indeed, under iron restriction, PerR-repressed genes should already be highly expressed and thus might not be responsive to H_2_O_2 _exposure. In contrast, the presence of iron promotes the production of damaging hydroxyl radicals through the Fenton reaction, and leads to severe oxidative stress. Consequently, it is not surprising that the transcriptional response to H_2_O_2 _exposure is dependent on the level of iron in the growth medium. Several interesting outcomes are worth discussing. First, of the 15 genes with altered transcription unique to H_2_O_2 _in absence of iron, eight (*aroC*, *chuB, chuC*, Cj0173, Cj0177, Cj1383, *exbB1 *and *tonB1*) are repressed by Fur and H_2_O_2_. This effect could be explained by H_2_O_2_-mediated release of iron from iron-sulfur clusters leading to elevated intracellular labile iron, which would elicit Fur repression. Second, the fold change of genes that are commonly affected by H_2_O_2 _is higher in the presence of iron, suggesting that these genes are regulated by both iron and H_2_O_2_. This expression profile could be explained by the presence of trace amounts of iron in the iron-restricted growth medium and by the regulation of these genes by PerR. Indeed, all these genes are members of the PerR regulon, consistent with the proposed mechanism of PerR regulation. Third, 12 of the 15 genes that were uniquely affected by H_2_O_2 _in the presence of iron are PerR-regulated, also corroborating the mode of PerR regulation.

Two important conclusions can be drawn from this study. First, H_2_O_2 _likely induces iron release from iron-containing proteins, leading to Fur repression. Second, and as expected, the level of iron present in the growth medium affects the extent of H_2_O_2 _regulation; some genes were more responsive than others, likely through the modulation of PerR activity.

### Transcriptome changes in response to oxidant exposure under iron-restricted conditions

Exposure of *C. jejuni *to cumene hydroperoxide, menadione, or hydrogene peroxide under iron restriction appears to elicit three distinct transcriptional responses. A Venn diagram analysis of genes belonging to the three stimulons identified 12 genes with a transcriptional profile common to all three treatments, and 11, 12, and 8 genes whose transcription was uniquely altered by cumene hydroperoxide, menadione, and H_2_O_2_, respectively. Importantly, while the differences in the populations of altered genes among the three stimulons might indicate specific transcriptional responses to each oxidant, it is also possible that these differences result from different levels of oxidative stress induced by each oxidant. Nevertheless, there are two trends worth noting. First, of the genes belonging to the cumene hydroperoxide, menadione, and hydrogen peroxide stimulons, up to 75% are PerR regulated, indicating that PerR is the major regulator of the oxidative stress response (Figure 2). Second, the 12 genes common to all three oxidant stimulons are PerR-regulated, suggesting that PerR is responsive to all three classes of oxidants.

Of the genes that were not regulated by PerR, *Cj1669c*, *uvrC*, and *dnaA*, which are involved in DNA repair and metabolism, are of particular interest. Genes involved in DNA repair have been found to be up-regulated in response to oxidative stress in numerous bacteria, including *S. aureus *[42,43], *E. coli *[7], and *P. aeruginosa *[44]. Consistent with the induction of DNA repair genes, bacteria mutated in these genes exhibit increased susceptibility to oxidants [7]. The ability to repair oxidative stress-induced DNA damage is an important defense mechanism [7]. However, in *C. jejuni*, the expression of most DNA-repair genes was found to be unaffected by oxidant exposure. Moreover, two of these genes--*Cj1669c*, encoding a putative ATP-dependent DNA ligase involved in DNA replication and repair; and *uvrC*, encoding an exonuclease ABC subunit C involved in nucleotide excision repair--were even found to be slightly down-regulated in response to menadione or cumene hydroperoxide exposure. Significantly, expression of the chromosomal replication initiator protein, *dnaA*, was also down-regulated in response to oxidant exposure. The down-regulation of these key DNA replication and repair genes might indicate a growth arrest, and likely reflects the relatively limited ability of *Campylobacter *to deal with DNA damage compared to other bacteria. Indeed, *C. jejuni *lacks a classical SOS response [45], which coordinates the induction of stress responses and DNA repair [46,47]. This might explain the absence of induction of DNA-repair genes in response to oxidant exposure. Nevertheless, the down-regulation of the *dnaA *gene might provide *Campylobacter *with additional time to repair DNA and/or prevent excessive accumulation of errors.

Although genes responsive to a specific oxidant could be indicative of specialized detoxification pathway(s), no clear trend could be inferred from the list of differentially expressed genes. Nevertheless, a few genes are worth noting. In particular, the multidrug efflux gene, *cmeA*, and two genes encoding heat-shock proteins, *grpE *and *hrcA*, were found to be specifically up-regulated in response to cumene hydroperoxide exposure. The *cmeA *gene (*Cj0367c*) has been previously characterized and shown to encode the membrane fusion component of a resistance-nodulation-division-type efflux pump that, in addition to CmeA, contains CmeB and CmeC proteins [48]. Notably, the *cmeB *and *cmeC *genes were up-regulated by 2- and 1.5-fold, respectively, with corresponding Bayesian *p*-values of 4 × 10^-3 ^and 3 × 10^-2^. The CmeABC pump confers resistance to various antimicrobials and bile compounds [48]. The observed up-regulation of CmeABC in response to cumene hydroperoxide exposure suggests an additional role for these pumps in oxidative stress defense, particularly in organic hydroperoxide resistance. Similar resistance mechanisms have been observed in other bacteria, including *S. enterica *serovar Typhimurium, where the efflux pump component, SmvA, has been shown to contribute to paraquat resistance by extruding toxic substrates from the cytoplasm [49]. In *P. aeruginosa*, the multidrug efflux pump *mexGHI *is regulated by the superoxide anion regulator, SoxR, which is induced in response to superoxide anions [50]. Clearly, the role of efflux pumps in the oxidative stress defense mechanisms of *C. jejuni *warrants further characterization. Other cumene hydroperoxide up-regulated genes of interest include those encoding the putative heat shock regulator, HrcA, and the heat shock protein, GrpE. The up-regulation of these two heat shock proteins might contribute to the repair of damaged proteins. However, no changes in the expression of other genes that encode chaperones, chaperonins, or heat shock proteins were found.

### Characterization of oxidative stress defense system mutants

In addition to our Δ*fur *and Δ*perR *mutants, we constructed three isogenic mutants, Δ*katA*, Δs*odB *and Δ*ahpC*, and the double mutant, Δ*perR*Δ*fur*, to further characterize *C. jejuni *oxidative stress defenses. The contribution of these genes to oxidative stress resistance was assessed by disk inhibition assays, as described by others [15,30]. As shown in Table 1, the Δ*fur *mutant was more resistant to H_2_O_2 _and more sensitive to cumene hydroperoxide and menadione than the wild-type strain, indicating that Fur represses genes involved in H_2_O_2 _defense and activates genes involved in cumene hydroperoxide and menadione resistance. Complementation of the Δ*fur *mutant in trans with the *fur *gene restored the wild-type phenotype, confirming its role in oxidative stress defense. It should be noted that the Δ*fur *mutant is predicted to exhibit an increased iron uptake phenotype, which, in turn, might lead to increased production of hydroxyl radicals due to an excess of iron in the cytosol. While this possibility could explain the hypersensitivity of the Δ*fur *mutant to cumene hydroperoxide and menadione, it is in disagreement with the observed H_2_O_2_-resistance of the Δ*fur *mutant (Table 1), suggesting that Fur might play a direct role in repressing H_2_O_2 _defense genes.

In agreement with this interpretation, Fur has been shown to regulate *katA *expression [22]. The Δ*katA *mutant was found to be considerably more sensitive to H_2_O_2 _and slightly more sensitive to cumene hydroperoxide, differences that were statistically significant (*P *< 0.05, by ANOVA). Chromosomal complementation of the Δ*katA *mutant restored its sensitivity to both oxidants to wild-type levels. The phenotype of the Δ*katA *mutant is in agreement with the recent report from Bingham-Ramos and Hendrixson showing that a Δ*katA *mutant of *C. jejuni *81-176 was 10,000-fold more sensitive to H_2_O_2 _than the wild-type strain [51]. The slightly reduced ability of the Δ*katA *mutant to survive cumene hydroperoxide stress compared to the wild-type strain suggests that KatA might also exhibit organic hydroperoxidase activity. However, AhpC appears to be the major contributor to organic hydroperoxide resistance, as evidenced by the significant defect in cumene hydroperoxide resistance observed in the Δ*ahpC *mutant (Table 1). Interestingly, inactivation of *ahpC *increased H_2_O_2 _resistance, suggesting a compensatory effect, likely through the over-expression of genes whose products are involved in responding to this particular stress. H_2_O_2 _sensitivity and cumene hydroperoxide resistance were fully restored by complementation of the Δ*ahpC *mutation through chromosomal reinsertion of the native *ahpC *gene. In agreement with the observed H_2_O_2 _resistance of the Δ*ahpC *mutant, mutation of *ahpC *in *B. subtilis *[52]was also shown to increase H_2_O_2 _resistance. The same phenotype was also observed in *Xanthomonas campestris *[53] and *S. aureus *[30]. Most AhpC enzymes exhibit a high specificity for hydroperoxide substrates (relative to catalase) and are the major enzymes responsible for removal of low levels of H_2_O_2 _from cells [54]. Consequently, mutation of *ahpC *should lead to an increase in the level of cytoplasmic H_2_O_2 _and activation of the oxidative stress stimulon. This would result in an increase in *katA *expression, consistent with the observed increase in H_2_O_2 _resistance. Based on the phenotypic data, a similar phenomenon is likely to manifest in the *C. jejuni *Δ*ahpC *mutant through induction of *katA *expression by relief of PerR repression. Alternatively, an *ahpC *mutation could lead to the induction of the two recently characterized thiol peroxidase genes, *tpx *and *bcp*, which have been shown to participate in H_2_O_2 _detoxification [55].

Inactivation of *sodB *led to an impaired ability to survive H_2_O_2_, cumene hydroperoxide and menadione (Table 1). A complementation analysis of the Δ*sodB *mutant demonstrated that the resistance to cumene hydroperoxide could be rescued by inserting the *sodB *gene in trans; however, the resistance to H_2_O_2 _and menadione could not be restored to wild-type levels. This partial complementation could be due to altered expression of *sodB*, reflecting the fact that the complemented *sodB *gene was expressed under the control of the kanamycin resistance promoter. Accordingly, the levels of SodB might not be sufficient to fully alleviate the deleterious effects of H_2_O_2 _and menadione. Menadione is a generator of superoxide anions; thus, the menadione sensitivity of the *sodB *mutant is in agreement with the function of SodB as an enzyme that dismutates superoxide to H_2_O_2_. Intriguingly, the *sodB *mutation also led to sensitivity to H_2_O_2 _and cumene hydroxyperoxide, oxidants that are not detoxified by SodB. An explanation for this increased sensitivity is that a *sodB *mutant would likely accumulate endogenous superoxide anions produced during normal metabolism. These superoxide anions would then oxidize iron-sulfur complexes, releasing catalytic ferrous iron [6,7]. In the presence of H_2_O_2_, the accumulation of Fe^2+ ^would catalyze the Fenton reaction, leading to the generation of hydroxyl radicals, which would damage DNA [6,7]. In the presence of organic hydroperoxide, the accumulation of iron would catalyze the generation of alkoxyl radicals, which would initiate lipid peroxidation. As a consequence, the *sodB *mutant should be sensitive to H_2_O_2 _and cumene hydroperoxide due to increased metal-catalyzed ROS production.

### Role of oxidative stress defense systems in gut colonization

**Figure 4:**
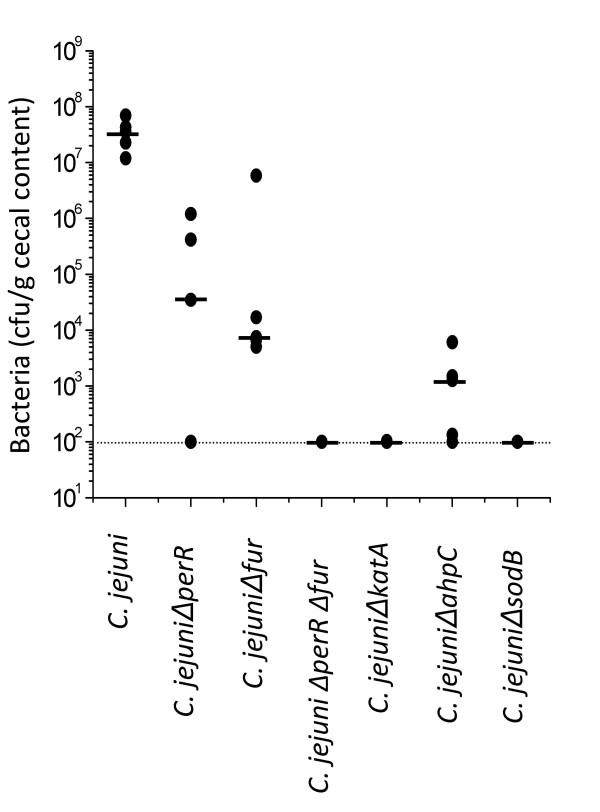
**Colonization levels of the *C. jejuni *wild-type and mutant strains in the chick model**. Groups of five chicks were inoculated with each *C. jejuni *strain. Each point represents the level of colonization of each strain from one chick. The bar indicates the median bacterial colonization level and the dashed line indicates the detection limit of the assay.

To examine the role of oxidative stress defenses in *C. jejuni *colonization, we tested *C. jejuni *NCTC 11168 and the Δ*perR*, Δ*fur*, Δ*perR*Δ*fur*, Δ*katA*, Δ*ahpC *and Δ*sodB *mutant strains in the chick animal model of colonization, as previously described [24,56]. As shown in Figure 4, Δ*perR*, Δ*fur*, Δ*perR*Δ*fur*, Δ*katA*, Δ*ahpC *and Δ*sodB *mutants exhibited a significantly altered ability to colonize the chick cecum compared to the wild-type strain (*P *< 0.05, by non-Parametric Mann-Whitney Rank-Sum test). Colonization of the chick ceca by the single-mutant Δ*perR *and Δ*fur *strains was reduced from the wild-type level of 2 × 10^7 ^cfu per gram of cecal content to 2 × 10^4 ^cfu/g and 5 × 10^3 ^cfu/g, respectively. Remarkably, the double mutation, Δ*perR*Δ*fur*, completely abolished the ability to colonize the chick ceca, reducing the level of colonization to below our detection limit (100 cfu/g). These findings suggest that iron homeostasis and oxidative stress regulation are important for survival and/or gut colonization. Mutations in *perR *and *fur *could have resulted in over-expression of attractive targets for chick antibodies, thus enhancing immune clearance. In addition, the motility defect of the Δ*perR *mutant might significantly contribute to the attenuated colonization behavior. Δ*katA *and Δ*sodB *single mutants were also unable to colonize the chick ceca (the number of mutants per gram of cecum was below the detection limit), and the colonizing ability of the Δ*ahpC *mutant was 50,000-fold lower than that of the wild-type strain. The colonization-defective phenotype of our Δ*ahpC *mutant in *C. jejuni *NCTC 11168 is more severe than that caused by the same mutation in *C. jejuni *81-176, which resulted in only a 10- to 50-fold reduction in chick colonization level compared to its respective wild-type strain [51]. This difference in colonization behavior between the two *C. jejuni *strains suggests the presence of an enhanced and/or additional AhpC-like activity in the *C. jejuni *81-176 strain that compensates for the effect of the Δ*ahpC *mutation. Collectively, these data provide definitive evidence that oxidative stress defense systems and associated regulatory mechanisms are important in *C. jejuni *colonization of the chick cecum and/or subsequent survival.

## Conclusion

**Figure 5:**
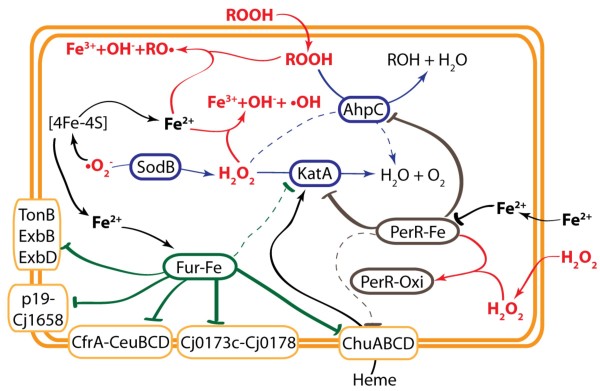
**Schematic representation of oxidative stress metabolism and regulatory network in *C. jejuni***. The oxidants are highlighted in red; the anti-oxidant proteins are boxed in blue; and the transcriptional regulators, Fur and PerR, are boxed in green and gray, respectively. SodB catalyzes the dismutation of superoxide anion (^.^O_2_) to hydrogen peroxide (H_2_O_2_). KatA reduces H_2_O_2 _to water and oxygen. AhpC catalyzes the reduction of organic hydroperoxide (ROOH) and H_2_O_2 _to their corresponding alcohols. Note that H_2_O_2 _reduction is primarily catalyzed by KatA (blue arrow) and secondarily by AhpC (dashed blue arrow). Intracellular iron reacts with H_2_O_2 _and ROOH to produce hydroxyl radicals (^.^OH) and alkoxyl radicals (RO^.^), the two most toxic ROS. Fe^2+^-Fur is the primary repressor of iron-acquisition pathways (green lines), including ChuABCD, and is the secondary repressor of KatA (dashed green line). Fe^2+^-PerR is the primary repressor of KatA and AhpC (gray lines), and the secondary repressor of ChuABCD (dashed gray line). H_2_O_2 _disrupts the ability of PerR to act as a repressor. Iron represses PerR through an unidentified pathway. Heme is transported by ChuABCD and is required for KatA activity.

We have described the transcriptional response of *C. jejuni *to H_2_O_2_, cumene hydroperoxide, and menadione exposure. Our results revealed that oxidants have a broad impact on genes involved in a variety of biological pathways, from classical oxidative stress defense systems and iron metabolism, to multidrug efflux pumps, heat shock proteins, DNA repair and metabolism, fatty acid biosynthesis, and membrane structure biogenesis. From this transcriptomic data, it is evident that *C. jejuni *has evolved complex mechanisms to cope with oxidative stress. Mutagenic, transcriptomic, and phenotypic studies of genes of interest revealed an interplay between the major players of the oxidative stress defense system--mainly the superoxide dismutase, SodB; the alkyl-hydroxyperoxidase, AhpC; the catalase, KatA; the ferric-uptake regulator, Fur; and the peroxide regulator, PerR--and the regulatory pathways that link them (Figure 5). The results presented in this study suggest that the level of intracellular iron is a key factor in the bacterial response to oxidative stress, reflecting the role of iron in catalyzing the formation of hydroxyl and/or alkoxyl radicals. A mutation in *ahpC *might indirectly up-regulate *katA *expression through relief of PerR repression, a compensatory response that would tend to restore H_2_O_2 _resistance. Interestingly, a mutation in *sodB *resulted in decreased resistance to H_2_O_2 _and cumene hydroperoxide. The accumulation of endogenously produced superoxide anions in the Δ*sodB *mutant would oxidize iron-sulfur clusters, releasing ferrous iron. The increase in ferrous iron would then catalyze the Fenton reaction, resulting in the decomposition of H_2_O_2 _and organic hydroperoxide, and the production of hydroxyl radicals and alkoxyl radicals, respectively. The hydroxyl radicals would damage DNA and proteins, whereas the alkoxyl radicals would initiate de novo lipid peroxidation [57]. Unexpectedly, the heme transporter ChuABCD of *C. jejuni *NCTC 11168 was found to be up-regulated upon H_2_O_2 _exposure in the presence of iron, but was down-regulated by H_2_O_2 _in the absence of iron. Under iron-restricted conditions, ChuABCD was expressed at a high level due to the absence of Fur repression. However, the addition of H_2_O_2 _(to iron-starved cells) would lead to the release of iron from iron-sulfur clusters that would interact with Fur, leading to *chuABCD *repression. Our transcriptomic studies (Figure 1) and our previous work [25] show that *chuABCD *is repressed by both Fur and PerR in the presence of iron. Under such conditions, the addition of H_2_O_2 _should deactivate PerR, relieving PerR repression and thus leading to *chuABCD *up-regulation. Up-regulation of the heme transporter in response to iron and H_2_O_2 _might allow for the increased uptake of heme that is required for the catalytic activity of KatA, and thus might enhance H_2_O_2 _detoxification. Clearly, these observations underscore the link between oxidative stress and iron metabolism, and highlight the interconnected regulatory network that coordinates this linkage. Finally, we have shown that oxidative stress defenses and the proper regulation of these defenses are essential for efficient colonization of the chicken cecum.

## Methods

### Bacterial strains, plasmids, and culture conditions

The bacterial strains and plasmids used in this study are listed in Table 3. All *C. jejuni *strains were grown at 37°C under microaerophilic conditions (83% N_2_, 4% H_2_, 8% O_2_, and 5% CO_2_) in Mueller-Hinton (MH) broth, MEMα medium, or on MH-agar plates. Media were supplemented as required with chloramphenicol (20 μg/mL) or kanamycin (10 μg/mL). Plasmids were constructed and propagated in *E. coli *DH5α or *E. coli *K-12. All *E. coli *strains were grown at 37°C in Luria-Bertani (LB) broth or on LB-agar plates. As appropriate, chloramphenicol or kanamycin was added to the *E. coli *LB medium at a concentration of 10 μg/mL or 50 μg/mL, respectively.

**Table 3:** Bacterial strains and plasmids used in this study.

**Strain or plasmid**	**Relevant characteristics ^*a*^**	**Source or reference**
*E. coli *strains		
DH5α	*end*A1, *hsd*R17 (rK^-^, mK^-^), *sup*E44, *thi*-1, *rec*A1, *gyr*A, *rel*A1, Δ(*lac*ZYA*arg*F)U169, *deo*R, *[f80dlac*Δ*(lacZ0 M15]*	Invitrogen
*E. coli *K-12	*end*A1, *hsd*R17 (r_K12 _^-^, m_K12 _^+^), *sup*E44, *th*i-1, *rec*A1, *gyr*A96, *rel*A1, lac F' [*proA*^+^*B*^+^, *lacI*^q^ZD*M*15::Tn 10(tet^R^)]	BD Fusion-blue competent cells
*C. jejuni *strains		
NCTC 11168	*C. jejuni *NCTC 11168	NCTC
AS230	NCTC 11168Δ*fur*	[24]
AS216	NCTC 11168Δ*perR*	This study
AS232	NCTC 11168Δ*fur*Δ*perR*	This study
AS433	NCTC 11168Δ*katA*	This study
AS438	NCTC 11168Δ*ahpC*	This study
AS446	NCTC 11168Δ*sodB*	This study
AS960	NCTC 11168Δ*fur+fur*	This study
AS978	NCTC 11168Δ*katA+katA*	This study
AS1016	NCTC 11168Δ*ahpC+ahpC*	This study
AS990	NCTC 11168Δ*sodB+sodB*	This study
AS1038	NCTC 11168Δ*perR+perR*	This study
Plasmids		
pUC19	Cloning and suicide vector, Amp^r^	Biolabs
pILL600	Plasmid carrying *kan*	
pRR	Fragment of rRNA gene cluster cloned into pGEM-T Easy	B. Wren
pRRC	*cam *cassette cloned into pRR	B. Wren
pRRK	*kan *cassette cloned into pRR	This study
pAS214	pUC19 carrying Δ*perR*::*cam*	This study
pAS215	pUC19 carrying Δ*perR*::*kan*	This study
pAS431	pUC19 carrying Δ*katA*::*cam*	This study
pAS436	pUC19 carrying Δ*ahpC*::*kan*	This study
pAS445	pUC19 carrying Δ*sodB*::*cam*	This study
pAS930	pRRK carrying the *fur *gene	This study
pAS946	pRRK carrying the *katA *gene	This study
pAS1010	pRRC carrying the *ahpC *gene	This study
pAS985	pRRK carrying the *sodB *gene	This study

^*a *^*cam*, chloramphenicol resistance gene; Amp^r^, ampicillin resistant; *kan*, kanamycin resistance gene.

### Construction of knockout mutants of the perR, katA, ahpC, and sodB genes of C. jejuni NCTC 11168

*C. jejuni *NCTC 11168 mutants containing mutations in *perR*, *katA*, *ahpC *or *sodB *genes were constructed using a mutagenesis and gene-replacement strategy. Briefly, the gene to be mutated was amplified by PCR from the chromosomal DNA of *C. jejuni *NCTC 11168 using the primers listed in Table 4 and high fidelity Pfx DNA polymerase (Invitrogen). The PCR products were digested with the appropriate restriction enzymes and cloned into pUC19 using a unique restriction site (Table 4). Deletions of 284, 392, 294, and 566 bp were generated by inverse PCR within *perR*, *katA*, *ahpC*, and *sodB *genes, respectively. The kanamycin resistance cassette of pILL600 was inserted into the deleted *ahpC *gene using a unique restriction site (Table 4), resulting in plasmid pAS436. The chloramphenicol resistance cassette from pRY111 was inserted into the deleted *perR*, *katA*, and *sodB *genes using a unique restriction site (Table 4), yielding pAS214, pAS431, and pAS445, respectively. The transcriptional orientation of the chloramphenicol and kanamycin cassettes was determined by DNA sequencing, and constructs in which the resistance cassette was in the same orientation as that of the mutated genes were used for natural transformation, as previously described [24,58]. The four mutants containing the corresponding suicide plasmid integrated into the chromosome of *C. jejuni *NCTC 11168 were obtained by selecting for kanamycin or chloramphenicol resistance. The identity of the mutants was confirmed by PCR analysis of the recombinants using the corresponding sets of primers.

**Table 4:** Primers used in this study.

**Primer**	**DNA sequence from 5' to 3'**
For gene cloning	
*perR-01*	ATGC**AGATCT**GTTATGGACAAGGTGTGGCA (BglII)
*perR-02*	ATGC**AGATCT**CATTGGAACTATCCAAAGTTGG (BglII)
*katA-01*	ATGC**GGTACC**CGATTTTGGAAACATTATAGCTGA (KpnI)
*katA-02*	ATGC**CTGCAG**TAATGGGGTTTGTCCACGAT (PstI)
*ahpC-01*	ATGC**AGATCT**CTGGAGTGTCCCCACTTCTC (BglII)
*ahpC-02*	ATGC**AGATCT**CCAAAACCCCAGCAATCATA (BglII)
*sodB-01*	ATGC**GAATTC**TTAAAAGTGGCCCATTCGTC (EcoRI)
*sodB-02*	ATGC**CTGCAG**TCCTTTTACTTCACGCAAGC (PstI)
For inverse PCR	
*perR-03*	ATGC**GGATCC**CACATAGTCTTTGCGGAGTAGC (BamHI)
*perR-04*	ATGC**GGATCC**GATAGGCAATCTCGTCAATCA (BamHI)
*katA-03*	ATGC**GGATCC**ATAAGCTCGGCAGCTTCTTG (BamHI)
*katA-04*	ATGC**GGATCC**ACAATGTCGCTGGTGCTATG (BamHI)
*ahpC-03*	ATGC**GGATCC**ACCGCTCCTTTTGGACCTAT (BamHI)
*ahpC-04*	ATGC**GGATCC**TCGCCATGCTGTGGTTAAT (BamHI)
*sodB-03*	ATGC**GGATCC**TCACCAAAAGCATTGGTATCAT (BamHI)
*sodB-04*	ATGC**GGATCC**GGCATGGGATCAGTTAGCTT (BamHI)
For complementation	
*aphA3*-01	TCC**CCCGGG**GATAAACCCAGCGAAC (SmaI)
*aphA3*-02	TCC**CCCGGG**AAGCTT**TCTAGA**CATCTAAATC (SmaI, XbaI)
*fur-05*	GATTTAGATGTCTAGCATGCTAGTGAAAAGTTGCAAGA
*fur-06*	GGGGAAGCTTTCTAGGCTTTTTCTATTCTTTGCTGCTC
*katA-05*	GATTTAGATGTCTAGTTACGTGCATCCCAGTGTTC
*katA-06*	GGGGAAGCTTTCTAGCCACCAAAAGTGGCAAGTAAA
*ahpC-05*	ATCCACTAGTTCTAGCATTCAACGCATTTGTTTGC
*ahpC-06*	CTAGGGCCGCTCTAGAAAAATGAAGAAAAAGCCTCGT
*sodB-05*	GATTTAGATGTCTAGTTTTTCATATTTTCCTCCTTATGAA
*sodB-06*	GGGGAAGCTTTCTAGCATTTTTGCCCCTTTTTGTG
*perR-05*	GATTTAGATGTCTAGGACCTATTGCTTTGCGTTAT
*perR-06*	GGGGAAGCTTTCTAGTGCTTAATGACACTTTTTGC
For identification of the rRNA insertion site	
Ak233	GCAAGAGTTTTGCTTATGTTAGCAC
Ak234	GAAATGGGCAGAGTGTATTCTCCG
Ak235	GTGCGGATAATGTTGTTTCTG
AR56	CATCCTCTTCGTCTTGGTAGC
Ak237	TCCTGAACTCTTCATGTCGATTG

The restriction sites used for cloning are bolded and the restriction enzymes used are indicated in parentheses.

### Mutant complementation

Knockout mutants of the *fur *(previously constructed [24]), *perR*, *katA*, *ahpC*, and *sodB *genes were complemented by insertion of the wild-type gene into the chromosome following the methodology developed by Karlyshev *et al. *[29], using either the pRR-Km [58] or pRR-Cm plasmid [29]. The five genes were amplified from the genomic DNA of *C. jejuni *NCTC 11168 using the proofreading Pwo DNA polymerase (Roche) and the respective primers listed in Table 4. Primers were designed to incorporate a 15-bp region of homology with each end of the XbaI-restricted pRR-Km or pRR vector. The *fur*, *perR*, *katA*, and *sodB *PCR products were cloned into pRR-Km using the In-Fusion PCR cloning kit from Clontech following the manufacturer's recommendations. The PCR product of *ahpC *was similarly cloned into pRR-Cm. The resulting plasmids were sequenced to confirm the absence of mutations and transformed into each respective mutant to construct the complemented strain. DNA transformation was performed as previously described [24,56,58], and transformants were selected on MH agar plates containing both kanamycin (10 μg/mL) and chloramphenicol (20 μg/mL). Finally, the chromosomal insertion of each gene into the rRNA locus was confirmed by PCR analysis using the primers listed in Table 4, as previously described [58].

### Disk inhibition assays

The *C. jejuni *NCTC 11168 wild-type, mutant and complemented strains were grown to mid-log phase in MH medium. The bacteria were harvested by centrifugation and resuspended in MH medium to an optical density of 1.0 at 600 nm. One milliliter of this bacterial suspension was added to 24 mL of melted MH agar, poured into Petri dishes, and allowed to solidify. Subsequently, 10 μL of either an aqueous 3% H_2_O_2 _solution, 3% cumene hydroperoxide in DMSO, or 90 mM menadione sodium bisulfite in H_2_O was pipetted on top of a 6-mm diameter disk in the center of the plate. Plates were incubated for 28 hours at 37°C under microaerophilic conditions. The zones of growth inhibition were determined by measuring the diameter of the clear zone, in millimeters. The disk inhibition assay was repeated at least three times for each strain, and the data was statistically analyzed using single-factor analysis of variance (ANOVA). A *P*-value < 0.05 was considered significant.

### Chick colonization assays

Cecum colonization was determined in a *C. jejuni *chick colonization model using 1-day-old pathogen-free broiler chicks, as described previously [24,56]. Briefly, five chicks were inoculated with 1.5-7 × 10^3 ^viable *C. jejuni *(wild-type or mutant strains). Four days later, the birds were humanely euthanized and necropsied. The ceca were collected, and their contents were homogenized, serially diluted in sterile phosphate-buffered saline buffer, and plated onto *Campylobacter *agar base (Oxoid CM935) containing *Campylobacter*-selective Karmali supplements (Oxoid SR167E). The plates were incubated for 48 hours at 37°C under microaerophilic conditions. The bacterial counts were determined and expressed as log_10 _of cfu per gram of ceca. A non-parametric Mann-Whitney Rank-Sum test was used for statistical analysis of the data. *P *values ≤ 0.05 were considered statistically significant.

### Extraction of total RNA

*C. jejuni *NCTC 11168 cells were incubated overnight with stirring at 37°C under microaerophilic conditions in a 500 mL flask containing 250 mL of MEMα medium. At mid-log phase, 50 mL of the culture was dispensed into 100 mL flasks and kept under microaerophilic growth conditions. To investigate the transcriptional responses of *C. jejuni *to oxidant exposure, H_2_O_2_, cumene hydroperoxide (CHP), or menadione sodium bisulfite (MND) was added to the 50 mL broth at a final concentration of 1 mM. The concentration of 1 mM was chosen because, unlike higher concentrations (e.g., 5 mM), it does not affect cell viability. The stock solutions of H_2_O_2 _and MND were prepared in H_2_O at a concentration of 0.882 M and 1 M, respectively, whereas the stock solution of CHP was prepared in DMSO at a concentration of 0.578 M. An equivalent amount of H_2_O or DMSO was added to the bacterial cultures that served as reference samples for the transcriptional profiling of responses to H_2_O_2_, MND, or CHP. Transcriptional responses of *C. jejuni *to H_2_O_2 _exposure in the presence of excess iron were investigated by adding ferrous sulfate to the bacterial culture to a final concentration of 40 μM 15 minutes prior to H_2_O_2 _exposure. Ten minutes after the addition of the oxidant, 25 mL of cells was mixed with 2.5 mL cold RNA degradation stop solution (10% buffer-saturated phenol in ethanol) [59]. The cells were pelleted by centrifugation and processed for total RNA extraction using a hot phenol-chloroform protocol, as described previously [24].

The PerR regulon was identified by first growing the wild-type *C. jejuni *NCTC 11168 strain and the Δ*perR *mutant in 500 mL flasks containing 250 mL of MEMα medium. At mid-log phase, 50 mL of the cultures were transferred to 100 mL flasks, and ferrous sulfate and/or H_2_O_2 _were added, as described above. Ten minutes after the addition of H_2_O_2_, the cells were collected and total RNA was extracted, as described above.

All RNA preparations were purified using the RNeasy kit (Qiagen, Valencia, CA), then treated twice with DNaseI (Invitrogen) and tested for the absence of genomic DNA by PCR. RNA integrity was evaluated by agarose gel electrophoresis, and RNA concentration was quantified using the RiboGreen RNA quantification reagent (Molecular Probes).

### Probe labeling and microarray hybridization

Fluorescently labeled cDNA was prepared as described previously [24]. In brief, 16 μg of total RNA was reverse transcribed in a total volume of 40 μL using Superscript II (Invitrogen) in the presence of 0.5 mM each of dGTP, dATP and dCTP, 0.16 mM dTTP, 0.34 mM aminoallyl-dUTP, and 10 μg of random hexamers. After incubating 2 hours at 42°C, the reaction was stopped and the RNA was destroyed by adding 4 μL of 50 mM EDTA and 2 μL of 10 N NaOH, and incubating at 65°C for 20 minutes. After neutralizing with 4 μL of 5 M acetic acid, the amino-modified cDNA was purified using a Microcon YM-30 filter (Millipore) according to the manufacturer's instructions. Next, the amino-modified cDNA was concentrated under vacuum using a SpeedVac and resuspended in 10 μL of 0.1 M sodium carbonate buffer (pH 9). The amino-modified cDNA was labeled with indocarbocyanine or indodicarbocyanine dye (Amersham), as previously described [24]. The fluorescently labeled cDNA was purified using QIAquick PCR spin columns according to the manufacturer's instructions (Qiagen, Valencia, CA). Differentially labeled cDNAs derived from *C. jejuni *reference cultures and cells subjected to oxidant, or from *C. jejuni *wild-type and Δ*perR *mutant, were pooled and hybridized to the *C. jejuni *NCTC 11168 microarray. The construction and validation of the *C. jejuni *microarray have been previously described [24,60]. This microarray consists of PCR products corresponding to approximately 98% of the predicted open reading frames identified in the first annotation of the *C. jejuni *NCTC 11168 genome [60]. The labeled cDNA pool was dried under vacuum and dissolved in 36 μL of hybridization buffer (5× SSC buffer [1× SSC: 0.15 M NaCl, 0.015 M sodium citrate, pH 7] containing 0.1% SDS, 25% formamide, and 25 μg salmon sperm DNA). The hybridization was performed under a cover slip using microarray slides pre-hybridized by incubating at 42°C for 45 minutes in 5× SSC buffer containing 0.1% SDS, 25% formamide, and 1% bovine serum albumin. After incubating for 18 hours at 42°C in a humidified chamber (Arrayit, Sunnyville, CA), the microarray slides were washed consecutively at room temperature in 2× SSC containing 0.1% SDS for 5 minutes and 0.1× SSC containing 0.1% SDS for 10 minutes, and then four times in 0.1× SSC for 1 minute. Finally, the slides were washed with distilled water, dried by centrifugation, and scanned with a laser-activated confocal scanner (ScanArray Gx, Perkin Elmer) at a resolution of 10 μm.

### Microarray data analysis

The signal intensities of each spot were collected using GenePix Pro4 software (Axon Instruments, Foster City, CA). Spots within regions of printing or hybridization anomalies were excluded from the analysis. The raw fluorescence intensity values were background subtracted, and spots with background-subtracted values less than three times the standard deviation of the local background in both channels were also excluded from further analysis. Subsequently, the background-subtracted fluorescent intensity in each wavelength (channel 1 for indodicarbocyanine [Cy5] and channel 2 for indocarbocyanine [Cy3]) was normalized using the MIDAS software (available from TIGR, ) [61] and by applying a locally weighted linear regression (Lowess), as previously described [24]. Microarray data from two independent biological experiments were collected, yielding six measurements per gene (three technical and two biological replicates) for each growth condition tested. Finally, the ratio of channel 2 to channel 1 was converted to log_2_, and the data were statistically analyzed using the empirical Bayes method, as previously described [24]. Genes were grouped based on their expression profile by hierarchical clustering analysis using the Genesis software (available from Graz University of Technology, Graz, Austria; ) [62]. All microarray data have been deposited in the NCBI Gene Expression Omnibus database under the accession number GSE13126. The microarray expression data for *ahpC*, *katA*, *sodB*, *cft*, and *perR *were confirmed by qRT-PCR using the QuantiTect SYBR Green RT-PCR kit, as previously described [24]. Quantitative fold changes were obtained using the comparative threshold cycle (ΔΔC_T_) method, as recommended by Applied Biosystems.

## Authors' contributions

KP carried out the transcriptomic studies, constructed the mutants, participated in the mutant phenotypic characterization, and drafted the manuscript. YS constructed the complemented mutants. AF participated in the mutant phenotypic characterization and carried out the motility assays. JB confirmed the microarray data using qRT-PCR and drafted part of the manuscript. HN participated to the chick colonization assays. AS conceived the study, participated in its design, performed the statistical analysis, and wrote the final manuscript. All authors read and approved the final manuscript.

## Supplementary Material

Additional file 1 **oxidant, Fur and PerR regulated genes**. The data provided represent the log in base 2 of the ratio of the treated cells, *perR *mutant or *fur *mutant to the reference cells. Click here for file

Additional file 2 **Fur regulated genes**. The data provided represent the log in base 2 of the ratio of the treated cells, *perR *mutant or *fur *mutant to the reference cells. Click here for file

Additional file 3 **PerR regulated genes**. The data provided represent the log in base 2 of the ratio of the treated cells, *perR *mutant or *fur *mutant to the reference cells. Click here for file

Additional file 4 **cumene hydroperoxide regulated genes**. The data provided represent the log in base 2 of the ratio of the treated cells, *perR *mutant or *fur *mutant to the reference cells. Click here for file

Additional file 5 **menadione regulated genes**. The data provided represent the log in base 2 of the ratio of the treated cells, *perR *mutant or *fur *mutant to the reference cells. Click here for file

Additional file 6 **hydrogen peroxide regulated genes**. The data provided represent the log in base 2 of the ratio of the treated cells, *perR *mutant or *fur *mutant to the reference cells. Click here for file

Additional file 7 **hydrogen peroxide regulated genes in presence of iron**. The data provided represent the log in base 2 of the ratio of the treated cells, *perR *mutant or *fur *mutant to the reference cells. Click here for file
